# Effects of GABA_A_ Receptor α3 Subunit Epilepsy Mutations on Inhibitory Synaptic Signaling

**DOI:** 10.3389/fnmol.2020.602559

**Published:** 2020-11-20

**Authors:** Parnayan Syed, Nela Durisic, Robert J. Harvey, Pankaj Sah, Joseph W. Lynch

**Affiliations:** ^1^Queensland Brain Institute, The University of Queensland, Brisbane, QLD, Australia; ^2^School of Health and Behavioural Sciences, University of the Sunshine Coast, Maroochydore, QLD, Australia; ^3^Sunshine Coast Health Institute, Birtinya, QLD, Australia; ^4^Department of Biology, Joint Center for Neuroscience and Neural Engineering, Southern University of Science and Technology, Shenzhen, China

**Keywords:** α3 subunit, GABA_A_ receptor, *GABRA3*, IPSC, missense mutation, epilepsy

## Abstract

Missense mutations T166M, Q242L, T336M, and Y474C in the GABA_A_ receptor (GABA_A_R) α3 subunit gene are associated with epileptic seizures, dysmorphic features, intellectual disability, and developmental delay. When incorporated into GABA_A_Rs expressed in oocytes, all mutations are known to reduce GABA-evoked whole-cell currents. However, their impact on the properties of inhibitory synaptic currents (IPSCs) is unknown, largely because it is difficult to establish, much less control, the stoichiometry of GABA_A_R expressed in native neuronal synapses. To circumvent this problem, we employed a HEK293 cell-neuron co-culture expression system that permits the recording of IPSCs mediated by a pure population of GABA_A_Rs with a defined stoichiometry. We first demonstrated that IPSCs mediated by α3-containing GABA_A_Rs (α3β3γ2) decay significantly slower than those mediated by α1-containing isoforms (α1β2γ2 or α1β3γ2). GABA_A_R α3 mutations did not affect IPSC peak amplitudes or 10–90% rise times, but three of the mutations affected IPSC decay. T336M significantly accelerated the IPSC decay rate whereas T166M and Y474C had the opposite effect. The acceleration of IPSC decay kinetics caused by the T366M mutation was returned to wild-type-like values by the anti-epileptic medication, midazolam. Quantification experiments in HEK293 cells revealed a significant reduction in cell-surface expression for all mutants, in agreement with previous oocyte data. Taken together, our results show that impaired surface expression and altered IPSC decay rates could both be significant factors underlying the pathologies associated with these mutations.

## Introduction

Type A γ-aminobutyric acid receptors (GABA_A_Rs) are pentameric ligand-gated ion channels found at a majority of inhibitory synapses in the central nervous system. They are anion-selective channels that mediate fast synaptic inhibitory neurotransmission on a millisecond timescale and are crucial for maintaining the excitatory/inhibitory balance of activity in the brain. GABA_A_Rs exhibit a vast heterogeneity, due to the large number of subunits which can combine in a myriad of combinations giving rise to unique subtypes. In humans, there are six α subunits, three β and three γ subunits, three ρ and one each of ρ, θ, δ and ε (McKernan and Whiting, 1996). Each of these subunits consists of a hydrophilic extracellular N-terminal domain containing a Cys-loop, followed by four α-helical transmembrane domains (TM1–4) and an extracellular C-terminus. TM2 lines the integral ion channel and the intracellular loop between TM3 and TM4 interacts with various proteins involved in receptor trafficking, phosphorylation, and clustering (Kasaragod and Schindelin, 2019).

The subunit composition of a receptor dictates its kinetic, pharmacological, and membrane surface localization properties. The α subunit, for example, determines isoform-selective pharmacology (Sieghart and Sperk, 2002), and synaptic localization of the receptor *via* direct interaction with gephyrin (Saiepour et al., 2010; Brady and Jacob, 2015; Gao and Heldt, 2016). Although the α1, α2 and α5 subunits have been extensively studied (Browne et al., 2001; Sieghart and Sperk, 2002; Jacob, 2019), relatively little is known about the α3 subunits. The α3 subunit is selectively distributed being primarily found at inhibitory synapses of the reticular thalamic nucleus (Pirker et al., 2000), the suprachiasmatic nucleus (Ono et al., 2018), and reelin-positive cells in the medial entorhinal cortex (Berggaard et al., 2019). It has also been shown to be expressed in the basolateral amygdala where it contributes to tonic inhibition (Marowsky et al., 2012). The α3 subunit is thought to be critical during brain development, as it is one of the most widely expressed α subunits in the brain during embryonic and early postnatal ages in the rat (Laurie et al., 1992; Wisden et al., 1992). It also undergoes A-to-I RNA-editing in the transmembrane domain, which may contribute towards synapse formation and maintenance of excitatory/inhibitory balance during development (Rula et al., 2008).

Alterations in GABAergic synaptic clustering, diffusion, or receptor kinetics can lead to several neurological diseases resulting from a loss of inhibitory control exerted by these receptors (Rudolph and Möhler, 2014). The study of disease-associated mutations in GABA_A_R subunit genes is important for understanding the role of individual GABA_A_R isoforms in the underlying pathogenesis of neurological disorders (Maljevic et al., 2019). This in turn can help us to understand the roles of particular isoforms under normal physiological conditions.

A recent study identified four missense mutations (T166M, Q242L, T336M, and Y474C) in the α3 subunit gene (*GABRA3*) that were associated with epileptic seizures, dysmorphic features, intellectual disability, and developmental delay (Niturad et al., 2017). All mutations were shown to substantially reduce total whole-cell currents for α3β2γ2S GABA_A_Rs expressed in *Xenopus* oocytes, while paradoxically increasing GABA sensitivity for the Q242L, T336M, and Y474C mutants. The genetic, clinical, and functional characteristics of the mutations are summarized in Table 1.

**Table 1:** γ-aminobutyric acid receptor (GABA_A_R) alpha3 subunit mutations.

cDNA	Protein	Mature protein	Inheritance	Domain	Artificial synapses	Surface trafficking	*Xenopus* oocytes^1^	Clinical features^1^
c.497C > T	**p.T166M**	**p.T138M**	**X-linked**	**ECD**	**Average decay significantly slower** (170 ± 15 ms) than WT (104 ± 9 ms) for α3β3γ2S	**75.6% decrease** in surface expression of α3β3γ2S	α3β2γ2S currents reduced by 75 ± 3%, decrease in protein by Western blotting	Generalized tonic-clonic seizures, intellectual disability, dysmorphic features including nystagmus (repetitive, uncontrolled eye movements), micrognathia (undersized lower jaw), arched palate, and delayed speech in two males. Absence seizures and learning defects in females.
c.725A > T	**p.Q242L**	**p.Q214L**	**X-linked**	**ECD**	**No significant effect** on any IPSC parameter for α3β3γ2S	**39.5% decrease** in surface expression of α3β3γ2S	α3β2γ2S currents reduced by 85 ± 3%, GABA sensitivity increased EC_50_ of 25 ± 2 μM	Pharmacoresistant epileptic encephalopathy (infantile or childhood onset), infantile spasms, tonic and generalized tonic-clonic seizures, moderate to severe ID and developmental delay in two males. Two affected females has a milder phenotype– treatable generalized tonic-clonic seizures, mild learning disability. All had micrognathia, short stature, dysmorphic features (e.g. cleft palate) and nystagmus.
c.1007C > T	**p.T336M**	**p.T308M**	**X-linked**	**TM2-TM3 loop**	**Average decay significantly faster** (59.9 ms ± 4.8 ms) than WT for α3β3γ2S. Midazolam **restored decay** to 111 ± 10 ms.	**47.4% decrease** in surface expression of α3β3γ2S	α3β2γ2S currents reduced by 91 ± 2%, GABA sensitivity increased EC_50_ of 38 ± 4 μM	Generalized tonic-clonic seizures, no reported additional morphological or behavioral symptoms in females.
c.1421A > G	**p.Y474C**	**p.Y446C**	*de novo*	**TM4**	**Average decay significantly slower** (147 ± 7 ms) than WT for α3β3γ2S	**56.3% decrease** in surface expression of α3β3γ2S	α3β2γ2S currents reduced by 68 ± 9%, GABA sensitivity increased EC_50_ of 22 ± 7 μM	Partial and tonic-clonic seizures and mild to moderate intellectual disability, other features including anxiety, speech defects and delayed language development in two females from different families.

*^1^Results from Niturad et al. (2017)*.

Importantly, the effects of the mutations on the properties of inhibitory synaptic currents (IPSCs) mediated by synaptic GABA_A_Rs is not known. Unfortunately, it is difficult to study individual, defined GABA_A_R isoforms in native neurons due to the multitude of other isoforms present, and the difficulty in pharmacologically or genetically isolating the isoform of interest at particular synapses. Also, *GABRA3* is subject to X-inactivation in all tissues except peripheral blood (Cotton et al., 2015), which may explain the variability in disease phenotype in males and females with a given GABA_A_R α3 mutations. X-inactivation is the process by which one of the copies of the X chromosome is randomly silenced in female cells during development. Males are expected to be hemizygous for *GABRA3* mutations (i.e., both α3 subunits in an α3β2γ2 heteropentamer will be mutant as they derive from a single X chromosome). However, female mutation carriers are likely to express either wild-type *or* mutant GABA_A_Rs (again with two mutant α3 subunits) in a mosaic pattern in different neurons in the brain, because X inactivation creates two populations of cells that differ in terms of the “active” X chromosome. To avoid these uncertainties and reproduce the mutant GABA_A_R subtypes found *in vivo*, we employed a HEK293 cell—neuron co-culture expression system that permits the recording of IPSCs from a pure population of GABA_A_Rs with a defined stoichiometry (Dixon et al., 2014, 2015).

## Materials and Methods

### Cell Culture and Transfection

HEK293AD cells were used for all electrophysiological experiments. The cells were cultured in monolayers in T75 flasks with Dulbecco’s Modified Eagle Medium (DMEM), supplemented with 10% fetal bovine serum. The cells were kept in a 5% CO_2_ incubator at 37°C and passaged at least once a week. Trypsinized cells from the flask were plated onto 35 mm dishes and transfected at 50–70% confluency using a calcium phosphate precipitation method. The transfected cells were incubated overnight in a 3% CO_2_ incubator with the transfection mix for 16–20 h and then washed with divalent cation-free phosphate-buffered saline (PBS) to terminate the transfection. The plasmids used were: human α1 (pcDNA3.1), human GABA_A_R β3 (pCMV6) and γ2S (pcDNA3.1), Neuroligin 2A (pNICE) and empty pEGFP. The wild-type (WT) human GABA_A_R α3 construct was generously provided by Dr. Philip Ahring (University of Sydney). Mutations were introduced using site-directed mutagenesis and confirmed *via* sequencing of the entire plasmid. The α, β and γ subunits were transfected with Neuroligin 2A and GFP at a ratio of 1:1:4:0.5:0.5 to optimize the incorporation of the γ subunit into triheteromers. Cells for immunocytochemistry were transfected in the same plasmid ratio but using Lipofectamine 2000 (Invitrogen) according to manufacturer instructions (total DNA:lipofectamine 1:1 ng/μl). In these experiments, transfected cells were incubated overnight in a 5% CO_2_ incubator with the transfection mix for 4–6 h and then washed with divalent cation-free PBS to terminate the transfection.

### Artificial Synapse Formation

Cortical neurons were harvested from Wistar rat embryos of both sexes at embryonic day 18 (University of Queensland, Institutional Breeding Colony). Euthanasia of timed-pregnant rats was performed via CO_2_ inhalation. All experiments were performed following relevant guidelines and regulations as approved by the University of Queensland Animal Ethics Committee (approval number: QBI/142/16/NHMRC/ARC). Cerebral tissue was extracted from the embryos as per protocol (Fuchs et al., 2013), and trypsinized using 0.25% trypsin-EDTA (Thermo Fisher, Australia). The tissue was triturated gently in DMEM and the neuronal suspension was centrifuged three times to maximize live-cell sedimentation. The cells were then counted and 70,000-80,000 neurons were plated onto 12 mm coverslips coated with poly-D-lysine in 4-well dishes. The medium was replaced with Neurobasal medium supplemented with 1% GlutaMAX and 2% B-27 24 h later, and half of it was again removed a week later and topped up with the freshly prepared medium. All components of the neuronal media were purchased from Thermo Fisher, Australia. Neurons were grown for 3–5 weeks at 37°C in a 5% CO_2_ incubator before being used for experiments. Transfected HEK293 cells were resuspended in the neuronal medium and plated onto neurons. The co-cultures were incubated overnight to allow for synapse formation and used over the next 1–3 days for electrophysiological recordings.

## Electrophysiology

Whole-cell patch-clamp electrophysiology experiments were performed at room temperature (20–23°C) on the transfected HEK293AD cells using an Axopatch 1D amplifier and pClamp10 software (Molecular Devices, San Jose, CA, USA). Cells were placed in a bath and continuously perfused with extracellular solution containing (in mM): 140 NaCl, 5 KCl, 2 CaCl_2_, 1 MgCl_2_, 10 HEPES, and 10 D-glucose, adjusted to pH 7.4 with NaOH. Patch pipettes, fabricated from borosilicate glass capillaries (Harvard Apparatus, Holliston, MA, USA), were pulled using a horizontal puller (Sutter Instruments, Novato, CA, USA), with the resistance of 3–6 MΩ, and fire-polished. The pipettes were filled with an intercellular solution containing (in mM): 145 CsCl, 2 CaCl_2_, 2 MgCl_2_, 10 EGTA, 2 MgATP, adjusted to pH 7.4 with CsOH.

Spontaneous GABAergic IPSCs were recorded at a holding potential of −70 mV; signals were filtered at 5 kHz and sampled at 20 kHz. Recordings with series resistance above 20 MΩ were discarded. The capacitance of the HEK293 cells was typically 20 pF, resulting in a typical corner frequency of 398 Hz. Because this was satisfactory for our experiments, series resistance compensation was not applied.

Midazolam (Sigma) was prepared in stock solutions of 10 mM in dimethylsulfoxide and stored at −20°C. Stock solutions were diluted to the desired concentration in extracellular solution on the day of recording. Typically, at least 3 min of spontaneous activity was recorded before and during drug application. To preserve network activity for spontaneous recordings, a drug solution was targeted to the recorded cell while the extracellular solution was washed over the surrounding area.

IPSC decay time constants, 10–90% rise times, and peak amplitudes were calculated using Axograph X (Axograph Scientific, Australia), as has been described previously (Dixon et al., 2015). Peak amplitudes were detected with a 2:1 signal to noise ratio as the threshold, and all peaks manually examined to select well-separated events. Parameters calculated by Axograph X for each event were averaged to determine the final values.

Statistical analysis and graph plots were performed using SigmaPlot 13 (Systat Software, San Jose, CA, USA). One-way ANOVA was used for group comparisons, and *p* < 0.05 taken to be statistically significant. Data are presented as mean ± SEM unless otherwise stated.

### Surface Labeling of Receptors

Unpermeabilized HEK293 cells were fixed with 4% paraformaldehyde 2 days post-transfection for 10 min and then washed with PBS and labeled with rabbit anti-GABA α3 antibody (Synaptic Systems), diluted 1:500 in blocking solution (1% bovine serum albumin) at 37°C overnight. Cells were then washed with PBS, incubated with goat anti-rabbit antibody conjugated with Alexa555 (Thermo Fisher Scientific) for 3 h, and mounted onto glass slides using DABCO (220 mM in 90% glycerol). Each isoform was tested across at least two separate transfections and immunocytochemistry sample preparation.

### Imaging and Analysis

All imaging was performed using the LSM 510 Meta inverted point-scanning laser confocal microscope (Carl Zeiss), fitted with a 63× 1.4 NA oil-immersion objective. The 488 nm laser was used to capture images of cells expressing GFP, and the 514 nm laser to visualize GABA_A_Rs containing the α3 subunit expressed at the cell surface. All exposure parameters were kept the same across all experiments to enable comparative levels of expression *via* fluorescence intensity measurement.

A custom code in ImageJ Macro language (IJM) was written in ImageJ (Fiji) to analyze and quantify the fluorescence from GABA_A_R α3 labelings for each cell (available on request). Global background subtraction was done for each image, and cells selected using regions of interest (ROI). The mean gray value of these ROIs was calculated and averaged. Mean, medians, and interquartile ranges were calculated across each dataset and one-way ANOVA on ranks used to determine statistical significance.

## Results

### Electrophysiological Characterization of Wild-Type α3β3γ2 GABA_A_Rs

**Figure 1:**
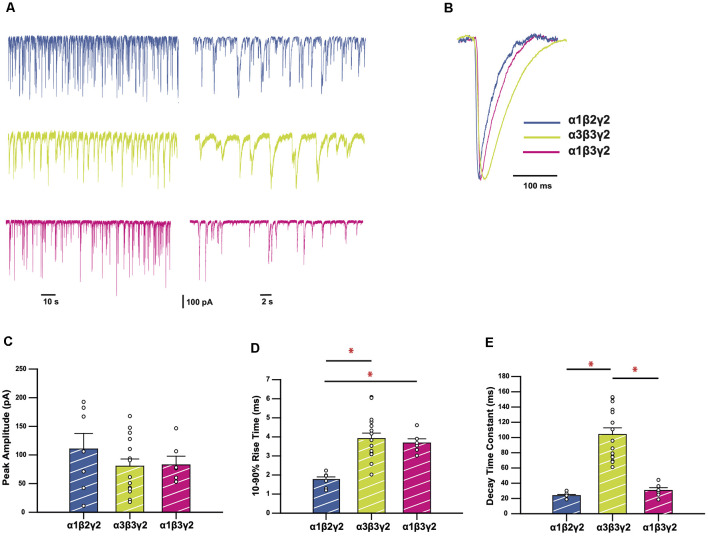
GABA_A_ receptors (GABA_A_Rs) containing the α3 subunit generate inhibitory synaptic currents (IPSCs) with slower rise times and decay time constants.** (A)** Representative traces recorded from HEK293 cells expressing the GABA_A_R isoforms as indicated. **(B)** Averaged and normalized IPSCs from individual cells, overlayed for comparison. **(C–E)** Bar plots comparing mean peak amplitudes **(C)**, 10–90% rise times **(D)**, and decay time constants **(E)** of wild-type (WT) GABA_A_R isoforms. Error bars represent SEM and *n* values were, respectively 7, 15, and 6. Statistically significant results (*p* < 0.05) are indicated with an asterisk.

We recorded spontaneous IPSCs in HEK293 cells expressing α3β3γ2 GABA_A_Rs, a physiologically-relevant receptor isoform found at central synapses (Fritschy and Mohler, 1995). We compared the kinetics of IPSCs mediated by this combination with those mediated by α1β2γ2, the most widely-expressed synaptic GABA_A_R subtype in the brain (Sieghart and Sperk, 2002). These results are summarized in Figure 1. Sample recordings shown on two different time-bases of IPSCs from HEK293 cells expressing wild-type (WT) receptors show markedly different decay kinetics for α1β2γ2 and α3β3γ2 GABA_A_Rs (Figure 1A). All isolated events from each of the recordings were averaged to produce a single digitally-averaged synaptic current waveform from each cell. These waveforms are shown normalized and superimposed in Figure 1B. The average peak IPSC amplitude, 10–90% rise time, and decay time constants from each cell are displayed in Figures 1C–E, where each data point represents the average from all well-isolated IPSCs recorded from a single cell. The comparison of α3β3γ2 with α1β2γ2 showed a significantly faster mean rise time (3.96 ± 0.29 vs. 1.76 ± 0.14 ms) and decay time constant (104.0 ± 8.7 vs. 23.7 ± 1.5 ms) for α1 containing receptors, whereas their respective IPSC amplitudes varied widely and were not significantly different to each other. To determine the contribution of β3 to these differences, we also compared these properties against those of α1β3γ2. These results (Figure 1), show that although the rise time of α1β3γ2 was also slow (3.68 ± 0.22 ms) compared to α1β2γ2, the decay time of α3-containing receptors was much slower than α1β3γ2 receptors (30.5 ± 3.6 ms).

### Effects of GABA_A_R α3 Subunit T166M, Q242L, T336M, and Y474C Mutations on IPSC Kinetics

**Figure 2:**
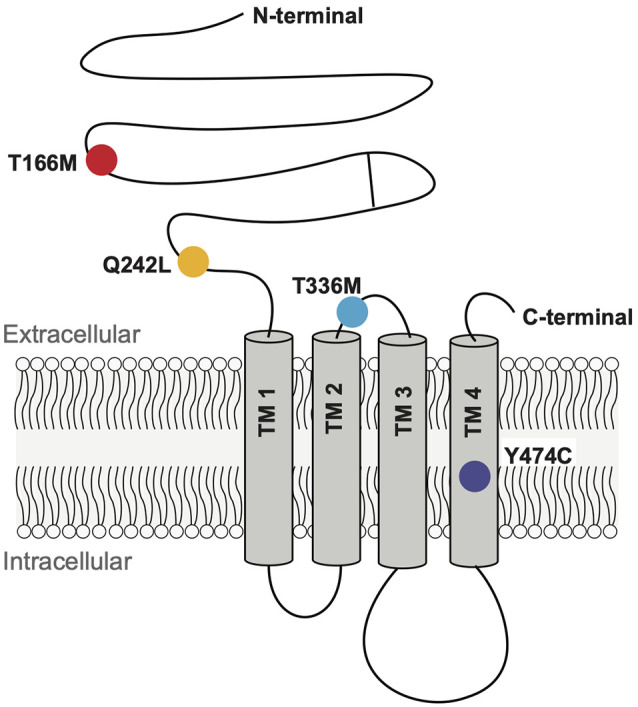
Schematic representation of a GABA_A_R subunit showing the positions of the four mutant residues investigated here.

We analyzed four GABA_A_R α3 subunit missense mutations (T166M, Q242L, T336M, and Y474C) that have previously been identified in patients with epileptic seizures, dysmorphic features, intellectual disability, and developmental delay (Niturad et al., 2017; Table 1). Their locations on the GABA_A_R α3 subunit polypeptide are shown in Figure 2. To understand the effect of these mutations we recorded IPSCs from co-culture synapses in which HEK293 cells had been transfected with the mutant GABA_A_R α3 subunits in combination with β3 and γ2 subunits.

**Figure 3:**
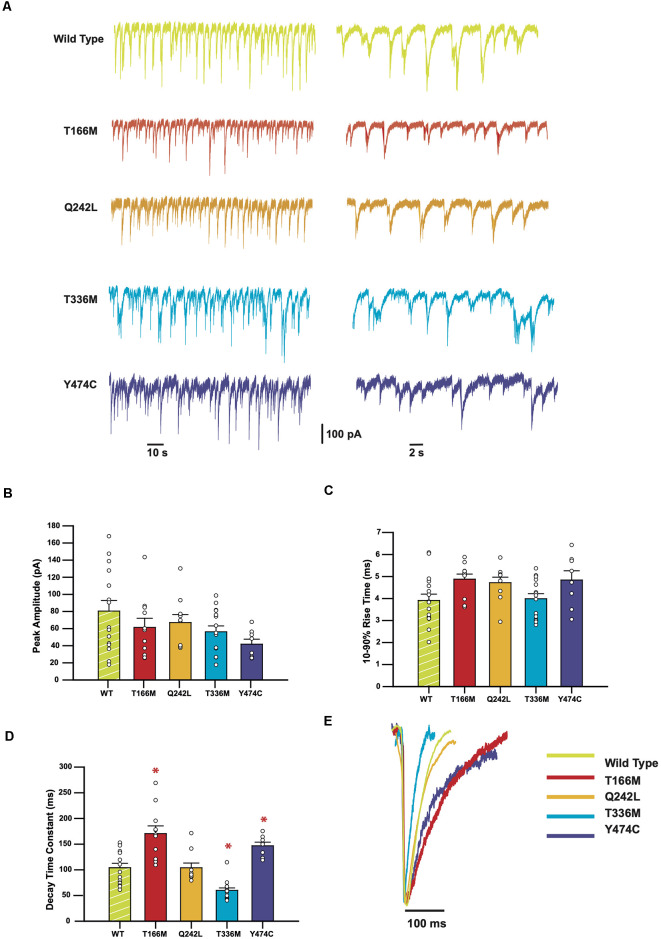
IPSCs mediated by GABA_A_R epilepsy mutants show a variety of changes in kinetic parameters. **(A)** Representative traces from HEK293 cells expressing WT or mutant α3 subunits, along with β3 and γ2. **(B–D)** Bar plots comparing the kinetic properties of the mutants compared to WT α3-containing GABA_A_Rs. Error bars represent SEM and *n* values were, respectively 15, 11, 9, 15, and 8. Statistically significant results (*p* < 0.05) are indicated with an asterisk. **(E)** Averaged normalized currents from single cells overlayed to compare the shape of the IPSCs recorded from each mutant compared to the WT.

Sample recordings of IPSCs shown on two different time-bases are shown in Figure 3A. Average peak IPSC amplitude, 10–90% rise time, and decay time constants from all events averaged from each cell are displayed in Figures 3C–E, where each data point represents the average of all IPSCs recorded from a single cell. The results indicate that while the average peak amplitudes and 10–90% rise times of all GABA_A_R α3 mutants showed no significant differences concerning WT receptors, the decay times of T166M and Y474C were significantly slower (170.6 ± 15.0 and 146.8 ± 7.1 ms respectively) than WT (104.0 ± 8.7 ms) whereas T336M was significantly faster (59.9 ± 4.8 ms). Q242L, on the other hand, had no significant effect on any measured IPSC parameter in these experiments. All these results are summarized in Table 1.

### Midazolam Treatment of T336M

**Figure 4:**
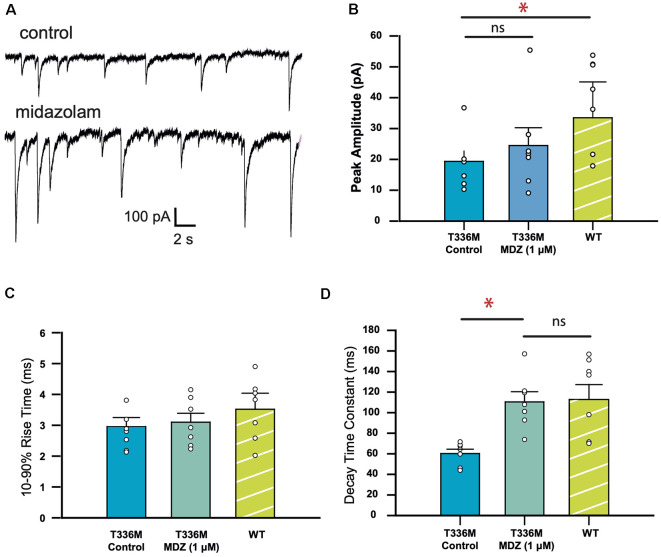
Midazolam restores the kinetic properties of GABA_A_Rs incorporating the GABA_A_R α3 subunit T336M mutation.** (A)** Representative traces recorded from a HEK293 cell expressing the T336M mutant, before and after midazolam treatment. **(B–D)** Mean kinetic properties of the T336M mutant before and after treatment with 1 μM midazolam. Control WT data are included for comparison (*p < 0.05; ns = no significant difference; *n* = 6 for all constructs).

Midazolam, a benzodiazepine, is a widely used anti-epileptic in infants and children that is thought to *act* via its action on GABA_A_Rs. Since the T336M mutation significantly increased the decay rate of IPSCs mediated by α3β3γ2 GABA_A_Rs, we tested whether the decay rate could be normalized using 1 μM midazolam. To account for variability between HEK293 cell batches, activity levels of neuronal cultures, and transfection efficiencies, the WT dataset was repeated under the same conditions for this set of experiments. A sample recording showing the effect of midazolam is shown in Figure 4A. Figure 4B shows that in the presence of midazolam, the average amplitude of IPSCs mediated by T336M-containing receptors increased from 19.3 ± 3.3 − 24.9 ± 5.7 pA, though this difference is not statistically significant and remains less than the WT average amplitude of 33.3 ± 5.0 pA. Similarly, the average IPSC rise time was not affected by midazolam (Figure 4C). However, the average decay time constant of IPSCs increased from 60.2 ± 4.2 − 110.5 ± 9.9 ms, which is comparable to the average decay time of WT α3β3γ2 GABA_A_Rs (108.1 ± 8.9 ms; Figure 4D). These results show that midazolam can restore the decay times of the T336M mutant GABA_A_Rs to values comparable with WT receptors.

### Surface Expression of the GABA_A_R α3 Subunit T166M, Q242L, T336M, and Y474C Mutants

Because of the varying effects of GABA_A_R α3 mutations on the kinetic properties of IPSCs and the complete lack of any observable effect for the Q242L mutation, we examined other mechanisms by which these mutations might be affecting receptor function. To this end, we used immunocytochemistry to quantify the surface expression for each mutant receptor and compared it to WT α3β3γ2 GABA_A_Rs. Transient transfection of transmembrane proteins in HEK293 cells often results in a large variation in surface expression even when the transfection is carefully optimized (Ooi et al., 2016). We, therefore, imaged large numbers of immunostained cells to obtain an accurate representation of the population. Exposure settings of the camera were kept constant throughout all experiments, and our inclusion criterion stipulated that any cell that showed an immunofluorescent signal discernible from the background would be included. In electrophysiology measurements, only cells with the highest levels of expression (judged by their fluorescence intensity) were selected for patching because those cells have the highest probability of forming synapses with the neurons and produce the most robust IPSCs.

**Figure 5:**
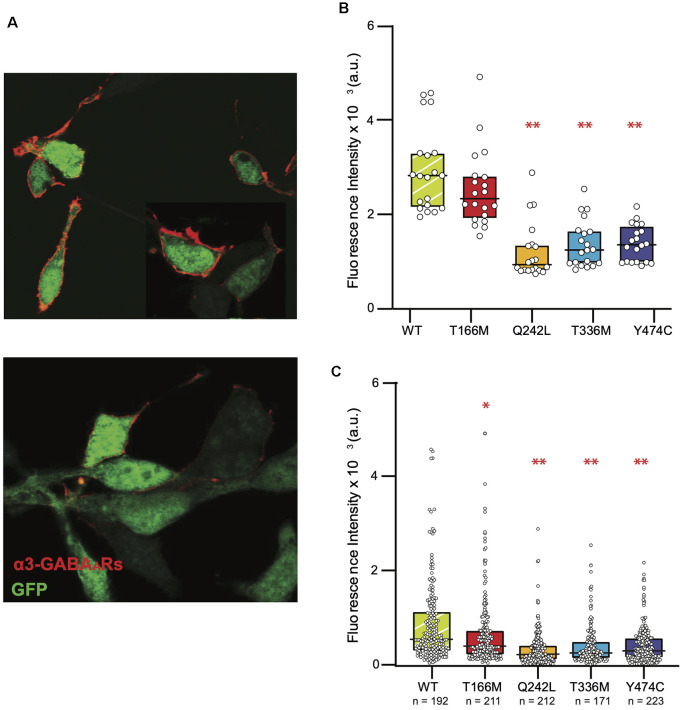
Reduced surface expression of mutant GABA_A_Rs in HEK293 cells. **(A)** Representative images of cells expressing α3β3γ2 receptors (red). Intracellular GFP (green) was used as an indicator of transfection efficiency. The top panel shows a sample image of cells showing a high level of expression and the bottom panel shows cells that had very low levels to no discernible expression levels. **(B)** Fluorescence intensity of the brightest cells for each isoform (*n* = 20 for all constructs) is shown as these cells are typically used to measure IPSCs in the “artificial synapse” system. The boxplots indicate the median and interquartile range, one-way ANOVA on ranks, **p < 0.001. **(C)** Full distribution of GABA_A_R surface expression in HEK293 cells shows reduced surface expression of all mutants compared to the WT, one-way ANOVA on ranks, *p < 0.05, **P < 0.001. Median (interquartile range) values are as follows: WT: 5.29 (3.01–11.4 × 10^2^) a.u., T166M: 4.0 (2.14–7.31) × 10^2^ a.u., Q2442L: 2.06 (1.05–4.19) × 10^2^ a.u., T336M: 2.51 (1.97–5.0) × 10^2^ a.u., Y474C: 2.98 (1.5–5.75) × 10^2^ a.u.

Representative images of cells expressing high and low levels of α3β3γ2 receptors are shown in Figure 5A. For comparison with electrophysiology measurements, we pooled twenty cells with the highest GABA_A_R surface expression from immunostaining experiments for the WT and each mutant (Figure 5B). The fluorescence intensity median (interquartile range), for WT α3β3γ2 was 28.2 (21.8–32.9) × 10^2^ a.u. All mutants had significantly lower fluorescence intensity, with median (interquartile range) values as follows: Q242L: 9.4 (8.2–13.4) × 10^2^ a.u., T336M: 12.5 (9.7–16.4) × 10^2^ a.u., and Y474C: 13.6 (9.9–16.8) × 10^2^ a.u. An exception was T166M: 23.3 (19.6–28.0) × 10^2^ a.u., which also trended towards lower expression than WT, but not to a statistically significant degree. However, when we included all imaged cells, we observed a significant decrease in the surface expression of all mutants with an overall reduction in median values to 75.6% for T166M, 39.5% for Q242L, 47.4% for T336M, and 56.3% for Y474C compared to WT α3β3γ2 (Figure 5C).

## Discussion

### GABA_A_Rs Containing the α3 Subunit Have Slow Decay Kinetics

This study aimed to elucidate the functional characteristics of α3β3γ2 GABA_A_R-mediated IPSCs and the effects of selected pathogenic mutations. α3-containing GABA_A_Rs are the main inhibitory synaptic receptors in the reticular thalamic nucleus, and are important for controlling the excitability of thalamocortical networks (Browne et al., 2001; Crabtree, 2018). However, IPSCs mediated by these receptors have not previously been studied in isolation. Inhibitory synapses within the thalamocortical network, like most GABAergic synapses in the brain, present heterogeneity in terms of subunit composition (Fritschy and Mohler, 1995). In the ventrobasal nucleus of the thalamus, α1-containing receptors mediate phasic inhibition and α4-containing receptors are involved in tonic inhibition (Jia et al., 2005). Studies of thalamocortical activity in α3-knockout mice have not been able to conclusively resolve the functional impact of these receptors, due to other compensatory mechanisms (Winsky-Sommerer et al., 2008; Schofield et al., 2009). In the present study, we used the HEK293-neuronal co-culture technique, because it allowed IPSCs mediated by a defined GABA_A_R isoform to be studied in isolation.

We demonstrated that IPSCs mediated by α3β3γ2 GABA_A_Rs decay slower than the other synaptically-abundant GABA_A_Rs, α1β2γ2 and α1β3γ2 (McKernan and Whiting, 1996; Hutcheon et al., 2004; Daniel et al., 2010). These results are in agreement with previously published studies that show that GABAergic IPSCs in the reticular thalamic neurons, which express mostly α3-containing receptors at synapses (Çavdar et al., 2014), are slower than those of neurons in the ventrobasal nucleus, where the α1-containing isoforms predominate (Schofield and Huguenard, 2007). It has also been shown in a single-channel study that α3-containing GABA_A_Rs have longer active periods, and higher intraburst open probabilities compared to α1-containing GABA_A_Rs, which results in slower deactivation of the channel (Keramidas and Harrison, 2010). The β3 subunit has also been shown to contribute to slow activation and decay kinetics of GABA_A_Rs in a co-culture system (Chen et al., 2017b). To rule out the contribution of the β3 subunit in the slow deactivation time of α3β3γ2 receptors, we also compared their IPSC kinetics with those of α1β3γ2 receptors. This analysis revealed that while the rise times are similar for the two β3-containing isoforms, α1β3γ2 receptors have decay times that are more comparable with α1- than α3-containing GABA_A_Rs. A comparison between all three receptor types shows that the α3 contributes to the slow IPSC decay time in a statistically significant manner.

### Effects of Pathogenic Mutations on IPSCs Mediated by α3-Containing GABA_A_Rs

Using the same methods, we tested how the mutations T166M, Q242L, T336M, and Y474C, identified in patients with epileptic seizures, dysmorphic features, intellectual disability, and developmental delay (Niturad et al., 2017) affected GABA_A_R mediated synaptic currents. These mutations did not affect average peak amplitudes or 10–90% rise time of synaptic currents, but all mutants, except Q242L, affected the decay time constants of the IPSCs (Table 1). The mutation Q242L, in the extracellular loop, associated with tonic-clonic seizures, dysmorphic features, intellectual disability, and developmental delay did not significantly affect any of the tested IPSC kinetic parameters. The mutant T336M, located in the TM2-TM3 loop, also associated with generalized tonic-clonic seizures, resulted in a significantly reduced IPSC decay time constant. This is similar to what has been observed for several epilepsy-related mutations in other GABA_A_R subunits (Fisher, 2004; Chen et al., 2017a; Dixon et al., 2017). The faster decay of the IPSC results in an overall loss of inhibitory control, which is likely to be the main cause of epilepsies associated with GABA_A_R mutations (Hernandez and Macdonald, 2019). A common pharmacological intervention for epilepsy is the use of benzodiazepines, which act on GABA_A_Rs and potentiate the influx of chloride ions (Ochoa and Kilgo, 2016). Midazolam is one such compound that has been used for the treatment of status epilepticus for decades (Smith and Brown, 2017). We tested whether this drug could restore the decay time of the mutant T336M GABA_A_Rs towards WT values, and found that indeed it does potentiate the mutant receptor to a level that could increase inhibitory control in the brain.

The mutations T166M in the extracellular domain, and Y474C in the TM4 domain, are associated with absence and complex partial seizures respectively, but also comorbidities such as autism, anxiety, dysmorphic features, and mild to moderate intellectual disability. Paradoxically, these mutations resulted in a slower decay time constant for the IPSCs. This has been previously observed for other epilepsy-causing mutants in GABA_A_R subunits, notably γ2 R43Q (Bowser et al., 2002), which is associated with febrile seizures and childhood absence epilepsy (Wallace et al., 2001). This apparent gain-of-function does not explain the associated pathology. However, further studies have shown that γ2 R43Q is retained in the endoplasmic reticulum (Durisic et al., 2018), resulting in reduced surface expression and synaptic targeting of the assembled receptor complexes (Frugier et al., 2007), which may explain the resulting loss of inhibitory control underlying the epileptic phenotypes.

These studies, as well as previous work on α3 mutations that demonstrated reduced whole-cell currents, and increased GABA sensitivity for Q242L, T336M, and Y474C (Niturad et al., 2017), led us to explore the effect of these mutants on cell-surface expression.

### Analysis of Cell-Surface Expression Efficiency

Our quantification experiments in HEK293 cells showed a significant reduction in surface expression for all mutants. This effect was most profound for mutation Q242L. This is corroborated by previous work in oocytes which showed reduced whole-cell currents for these mutants (Niturad et al., 2017). Decreased cell surface expression reduces overall inhibition, resulting in the manifestation of epilepsy phenotypes (Fisher, 2004; Durisic et al., 2018; Shi et al., 2019). However, it may also result in the preferential expression of receptors lacking the α3 subunit which could in turn alter IPSC kinetics. This is certainly a possibility in neurons where compensatory upregulation of other α subunits may occur. However, such a mechanism is unlikely to explain our results as HEK293 cells express no endogenous α subunits and functional synaptic receptors do not form without these.

Paradoxically, the peak amplitude of IPSCs recorded from co-cultures was not reduced in magnitude (Figure 3B). Thus, they do not reflect the observed differences in cell surface expression between the WT and mutant α3-GABA_A_Rs for two reasons. First, in co-culture preparations, we typically focused on cells with the highest expression level as those cells are most likely to form synapses with neuronal presynaptic terminals. While the highest expressing HEK293 cells allow for the recording of robust synaptic currents, they also attract multiple synaptic contacts (Leacock et al., 2018). As a result, electrophysiological recordings from these cells are typically characterized by overlapping events as seen in Figure 2A. These events, often with the largest amplitudes, are discarded in the analysis because they do not allow for accurate calculation of the rise and decay times. Thus, while cell-surface expression and IPSC amplitudes are expected to correlate linearly, the different analysis of the two datasets shows different trends and the expression levels may be underestimated when judged from the peak amplitude of IPSCs measured in highly-expressing cells.

Second, it is not known if the extracellular N-terminal domain of the α3 subunit is involved in synapse formation in HEK293 cells. While it has been shown that N-terminal domains of GABA_A_R α1, β2, and γ2 subunits are directly involved in synaptic contact formation (Oh et al., 2016; Lu et al., 2017), very little is known about the role of α3 subunit in synaptogenesis. Nevertheless, it is noteworthy that the GABA_A_R isoforms studied in these experiments did contain the γ2 subunit, which could potentially increase GABA_A_R clustering at postsynaptic densities (Schweizer et al., 2003), as has been shown for α1β3γ2 receptors (Brown et al., 2016). This would increase the IPSC peak amplitude even when the overall expression level in HEK293 cells is very small. Therefore, the direct correlation between the peak amplitude of IPSCs recorded from co-cultures and GABA_A_R surface expression levels may be lost.

Immunostaining experiments are unaffected by these factors and enable the detection of very low cell-surface expression levels, which results in a more accurate measure of GABA_A_R levels. Taken together, our results suggest that receptor trafficking to the cell surface could be a significant factor underlying the pathologies associated with these mutations. However, a detailed study examining the subcellular localization of these mutants and quantification of cell-surface expression levels using biotinylation or differential labeling is still warranted. Defects in receptor trafficking could be corrected using drugs such as SAHA, which can rescue misfolded proteins from the ER and restore expression levels of low-expressing GABA_A_R mutants (Chen et al., 2017a; Durisic et al., 2018). Patients carrying mutations like Q242L, which causes low expression levels without affecting channel kinetics, could potentially benefit from these types of therapies.

## Conclusion

This study reveals unique kinetic properties of IPSCs mediated by α3-containing GABA_A_Rs in a co-culture system, and shows that they have slower decay kinetics than other synaptic GABA_A_R isoforms. We also show that disease-causing mutations affecting the GABA_A_R α3 subunit have significant but varied effects on the functional properties of IPSCs mediated by α3-containing GABA_A_Rs. Of particular note, the acceleration of IPSC decay kinetics caused by the T366M mutation was returned to WT-like levels by the antiepileptic drug, midazolam. Finally, we showed that all mutations studied induced a significant reduction in cell-surface expression of GABA_A_ receptors, which indicates that effective pharmacotherapies should target deficient channel kinetics, whilst restoring the cell-surface expression of mutant subunits.

## Data Availability Statement

The raw data supporting the conclusions of this article will be made available by the authors, without undue reservation.

## Ethics Statement

The animal study was reviewed and approved by University of Queensland Animal Ethics Committee (approval number: QBI/142/16/NHMRC/ARC).

## Author Contributions

All authors contributed to experimental design. RH and P. Syed performed the molecular biology. P. Syed performed all experiments. P. Syed and ND analyzed the data. P. Syed and JL wrote the first draft of the manuscript. All authors were involved in revising the manuscript for important intellectual content, and all authors approved the final version to be published. All authors contributed to the article and approved the submitted version.

## Conflict of Interest

The authors declare that the research was conducted in the absence of any commercial or financial relationships that could be construed as a potential conflict of interest.
